# Regional cognitive pathways to intention to use AI: a structural equation modeling approach

**DOI:** 10.3389/fpsyg.2026.1758998

**Published:** 2026-05-20

**Authors:** Yewon Jang, Tae Gyoon Jeong, Tongjoo Suh

**Affiliations:** College of Natural Resources and Life Science, Pusan National University, Miryang, Republic of Korea

**Keywords:** AI application capability, AI concerns, AI knowledge, cognitive pathways, intention to use AI, perceived efficiency

## Abstract

The aim of this study was to examine how the cognitive pathways linking artificial intelligence (AI) knowledge and AI application capability to the intention to use AI differ across regions. Perceived efficiency and AI concerns are modeled as perceptual factors associated with AI adoption decisions. Using nationwide survey data from Korea, a multi-group structural equation model distinguishing between megacity and non-megacity regions was estimated. The results showed that perceived efficiency is closely associated with the intention to use AI, whereas AI concerns show a weaker association. In the megacity region, AI application capability was positively associated with perceived efficiency, whereas in the non-megacity region, both AI knowledge and AI application capability were positively associated with perceived efficiency and, in turn, the intention to use AI. These findings suggest that AI-related decision-making is associated not only with individual technical proficiency but also with contextual access to AI. Accordingly, policies should account for contextual disparities and consider strategies that strengthen AI knowledge and application capabilities in non-megacity regions to support more inclusive digital transformation.

## 1 Introduction

Modern society is undergoing a profound transformation across all sectors owing to advancements in artificial intelligence (AI). AI has the potential to increase productivity and wealth across industries and to replace or augment human labor, thereby significantly impacting business operations and employment (Makridakis, 2017). Such technological progress is recognized as a tool that assists human activity and dramatically enhances work efficiency.

As AI rapidly spreads throughout society, positive expectations for technological innovation coexist with social concerns (Chen and Lin, 2024), including privacy infringement and job displacement (Idoko et al., 2024). These attitudes differ depending on individual experiences and social contexts, and these perceptual differences directly influence the speed and direction of technological diffusion. Beyond technological advancements, questions have emerged regarding how individuals perceive and use AI.

The acceptance of AI represents not only an evaluation of its utility but also a cognitive and behavioral process shaped by individual evaluative responses, perceptions, and attitudes (Dagtekin and Kabakus, 2025). In this regard, AI acceptance simultaneously encompasses the positive aspects represented by perceived efficiency and the negative aspects reflected in concerns about AI. As AI diffuses across uneven regional information environments, disparities in AI literacy and application capabilities may fundamentally alter the formation of these cognitive pathways. The accessibility and integration of AI technologies frequently exhibit significant regional disparities (Farahani and Ghasemi, 2024). Such disparities may result in differences in exposure to technological environments and access to digital information resources. However, whether this perceptual pathway functions identically across different regions remains unclear.

In megacity regions, where individuals are more exposed to AI in practice, the extent to which they can use AI proficiently is likely to be a key determinant of perceived efficiency. Conversely, in a non-megacity region, where technological accessibility tends to be lower, the degree to which one understands AI conceptually may serve as a precondition for perceiving efficiency. In other words, the formation pathways of perceived efficiency may vary by region (Hwang, 2004); residents in megacity regions may recognize the usefulness of AI through actual work experience, whereas those in non-megacity regions may form perceived efficiency through both a conceptual understanding of AI and emerging practical capability. Thus, AI comprehension and practical use may have different implications across regions, and such regional differences in perceptual pathways may shape technology acceptance in distinct ways.

In addition, individuals’ awareness of the potential risks of AI can exacerbate AI concerns. Given that individuals in megacity and non-megacity regions experience different levels of accessibility to and practical engagement with AI, they are likely to vary across regions (Maghsoudi et al., 2025). Therefore, it is necessary to empirically examine how the relationships among AI knowledge, AI application capability, perceived efficiency, AI concerns, and intention to use AI differ across regional contexts.

The aim of this study was to empirically identify contextual variations in the cognitive pathways of AI-related decision-making across megacity and non-megacity settings, and provide insights for balanced technological adoption across regions. By comparing megacity and non-megacity regions, this study used structural equation modeling (SEM) to examine the associations between perceptual pathways and the intention to use AI. Specifically, the objectives were to (1) examine how individual AI knowledge and AI application capability are associated with perceived efficiency and AI concerns, (2) assess the direct and indirect effects of perceived efficiency and AI concerns on the intention to use AI, and (3) compare how these perception–behavior pathways differ between regions. Through this analysis, this study proposes directions for enhancing AI knowledge and acceptance in non-megacity regions, thereby contributing to the realization of inclusive digital transformation.

## 2 Literature review

### 2.1 Theoretical background

As the technological advancement paradigm shifts (Feuerriegel et al., 2024) and technology is recognized as a significant factor influencing social norms, discussions have increasingly focused on not only on the positive effects of technology but also on its social impact and responsibility (Hagendorff, 2020). In particular, with the expanding societal influence of AI, concerns about inherent risks, such as privacy infringement and algorithmic bias, have become central issues (Oseni et al., 2021; Kordzadeh and Ghasemaghaei, 2022). Therefore, understanding how individuals perceive the benefits and risks of AI is essential for sustainable AI adoption. Accordingly, balancing ethical regulations and technological innovation has emerged as a key challenge for a sustainable future. Furthermore, from the perspective of government agencies, approaches that simultaneously promote innovation and ensure safety have been emphasized under conditions of uncertainty (Ghosh and Lakshmi, 2023).

In addition to these social and institutional discussions, individual perceptions of and attitudes toward technology adoption have become crucial factors. For new technologies to diffuse throughout society, an understanding of how users perceive and accept them is fundamental. One of the most representative theories explaining this process is the technology acceptance model (TAM), based on the premise that beliefs and attitudes influence human behavior. It encompasses two key factors that affect the individual acceptance of information technology: perceived ease of use and perceived usefulness. These two factors shape users’ attitudes and intentions toward technology adoption and ultimately affect their actual use behavior. Perceived ease of use refers to the degree to which a user believes a system can be used with minimal effort, whereas perceived usefulness refers to the degree to which a user believes that using a particular system enhances workplace performance (Davis, 1985). Specifically, TAM explains that higher levels of perceived ease of use and perceived usefulness lead to more positive attitudes toward technology, which in turn influence behavioral intention to use the technology (Venkatesh and Davis, 2000). In the context of AI, expectations often focus on efficiency gains, such as saving time and increasing productivity. Accordingly, within this framework, perceived efficiency was examined as an evaluative belief related to both perceived usefulness and ease of use.

Since its introduction, TAM has been extended and applied in various studies, including numerous empirical studies on AI technology. Na et al. (2022) used TAM to explore the factors influencing the acceptance and intention to use AI-based technologies among construction firms. Choung et al. (2023) investigated the role and pathway characteristics of trust in shaping the intentions of university students and the general public to adopt AI voice assistants. Khan et al. (2024) applied a TAM-based model to examine human resource (HR) managers’ intentions to adopt AI technologies in HR management and proposed strategic directions for implementing AI-based HR systems. These studies indicate that research on AI acceptance has been steady. However, most studies focused on specific types of AI technologies, thereby limiting their generalizability. Therefore, the present study aimed to examine the intention to use AI from a more integrative perspective.

However, technology acceptance cannot be fully explained by individual attitudes or cognitive factors. Social and environmental factors, such as information accessibility and AI application capability, may act as preconditions for technology adoption. In this context, the concept of digital divide is particularly relevant. The digital divide refers to disparities in access to, use of, and proficiency in information and communication technologies arising from demographic, social, and economic differences (Hargittai, 2001), leading to broader social inequalities (Cullen, 2001). The fundamental reason for the emergence of the digital divide as a major social issue is that new value is created through information usage, and disparities in this capacity directly lead to social inequality (Ragnedda, 2017; Helsper, 2021). Among the main causes of the digital divide, differences in digital literacy levels have been most frequently emphasized. Digital literacy refers to the comprehensive ability to actively access various digital technologies and media, search for and understand information, and evaluate it critically (Bawden, 2008). It is considered an essential competence in the digital environment, and insufficient literacy can exacerbate both the digital divide and social inequality, owing to differences in the ability to utilize information (Helsper, 2016). Groups with low digital literacy, such as people with disabilities, low-income populations, farmers, fishermen, and older adults, are typically classified as information-vulnerable (Suh, 2025). These groups tend to exhibit relatively low levels of digital informatization due to economic constraints and limited access to information and communication infrastructure (Robinson et al., 2015; Friemel, 2016).

Within this broader context, this study focused on two distinct AI-related dimensions: AI knowledge and AI application capabilities. AI knowledge refers to the conceptual understanding of AI, including its principles, applications, and limitations. In contrast, AI application capability refers to the practical ability to use AI tools in problem-solving and task performance. These two dimensions may shape acceptance in different ways. In the case of AI knowledge, the direction of its effect on concerns is difficult to determine. For instance, while increased knowledge may mitigate concerns by reducing uncertainty, it may also intensify them by increasing the awareness of AI-related risks (Wei et al., 2025). In contrast to AI knowledge, the AI application capability implies a certain degree of self-selected adaptation to AI tools. Practice-based familiarity is likely to buffer psychological resistance (Yan et al., 2022). Therefore, AI application capability is expected to reduce AI concerns rather than intensify them.

Furthermore, the digital divide may influence individuals’ intentions to accept technological change, depending on their level of digital literacy (Helsper, 2012). The intention to use AI refers to the degree to which users are willing to adopt a particular technology or service and serves as a key determinant of actual technology use (Venkatesh et al., 2016). As AI increasingly integrates into daily life, groups with low digital literacy, including those with AI literacy, are more likely to perceive technological change as a threat and develop AI concerns (Polat, 2025; Li et al., 2025). In other words, the higher the capability to utilize technology, the lower the resistance to technological progress and the stronger the tendency toward learning and adoption.

### 2.2 Korean technological context

The foundation of its highly developed IT infrastructure has enabled South Korea to undergo rapid digital transformation and achieve widespread Internet penetration (Oh and Larson, 2011). Consequently, this process fosters a technology-receptive culture that facilitates active use of information (Shin and Koh, 2017). However, the metropolitan concentration of information technology has also contributed to a two-tier pattern of digital inequality in terms of Internet engagement, broadly separating Seoul from the rest of the country and raising the risk of the gap between Seoul, its strong infrastructural and cultural foundations, and less-advantaged regions becoming increasingly entrenched (Kim et al., 2025). The Seoul Metropolitan Area, centered on Seoul, accounts for more than half of South Korea’s total GDP and population, and this concentration has intensified further in recent years (Lee and Ishiro, 2023). Seoul’s economic structure has evolved from traditional manufacturing to knowledge-based services and advanced information and communication technology (ICT) industries (Kim and Han, 2012). In addition, more than 40% of Korea’s high-tech firms are located in Seoul, where key elements of the national innovation system, including elite universities, corporate R&D facilities, and venture capitalists, are highly concentrated, making the city the technological center of the Korean economy (Sohn and Kenney, 2007). These structural differences suggest that individuals in the Seoul metropolitan area are more likely to be exposed to advanced technological environments, which, in turn, provide greater opportunities for practical AI use and access to information resources (Hwang, 2004). In contrast, those in regions outside the Seoul metropolitan area may face relatively more limited conditions in these respects (Suh, 2025). Accordingly, the distinction between megacity and non-megacity regions in this study is intended to capture broader differences in the technological environment and opportunities for digital engagement rather than a purely geographic contrast.

## 3 Methods and data

### 3.1 Structural equation modeling

To identify the developmental direction local communities should pursue amid ongoing advances in science and technology, it is necessary to empirically examine the perceptual pathways and associations that shape the acceptance of AI technology. In the process of introducing and using new technologies such as AI, AI knowledge, and AI application capability are assumed to be significantly related to the perceived efficiency and AI concerns that emerge in the acceptance frame. These factors are expected to be associated with individuals’ intentions to use AI.

**Figure 1:**
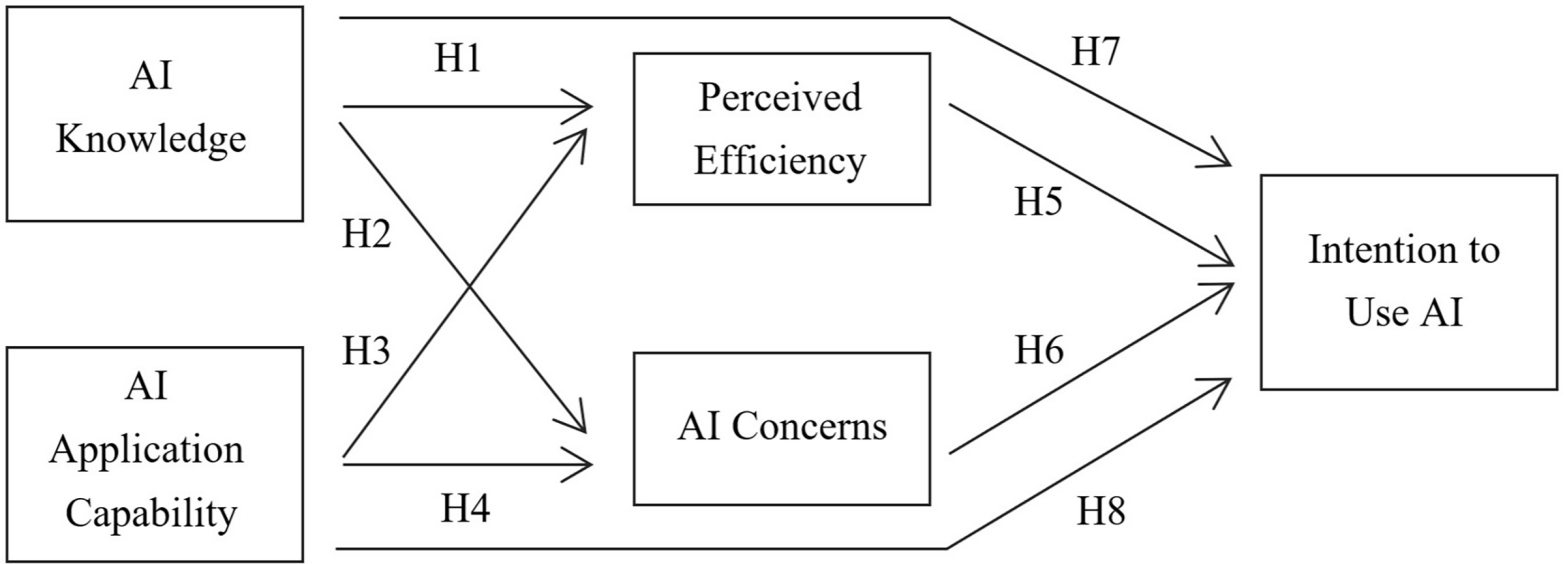
Conceptual framework of the hypothesized pathways. H1: AI knowledge positively influences perceived efficiency. H2: AI knowledge significantly influences AI concerns. H3: AI application capability positively influences perceived efficiency. H4: AI application capability negatively influences AI concerns. H5: Perceived efficiency positively influences intention to use AI. H6: AI concerns negatively influence intention to use AI. H7: AI knowledge has a direct effect on intention to use AI. H8: AI application capability has a direct effect on intention to use AI.

The aim of this study was to identify how individuals’ perceptions and application capabilities regarding AI are associated with their intention to use AI, with perceived efficiency and AI concerns included as perceptual variables in the path model. The following hypotheses were established based on this study; a conceptual model depicting the hypothesized pathways is shown in Figure 1.

To test these hypotheses, SEM was used to analyze the pathways associated with the intention to use AI. This approach is useful in that it enables the simultaneous estimation of multiple interdependent equations, including estimated direct and indirect effects, rather than relying on a sequence of separate regressions (Gunzler et al., 2013; MacCallum and Austin, 2000). Additionally, SEM can account for correlated disturbances among endogenous variables, which is important when cognitive evaluations (e.g., perceived efficiency and AI concerns) are jointly determined (Koopmans, 1945; Fox, 2002).

The structure of the model can be expressed as follows:

η=Πη+Γξ+ζ

where *η* represents the vector of endogenous variables, *ξ* is the vector of exogenous variables, *Π* is the matrix of path coefficients among endogenous variables, *Γ* is the coefficient matrix of the effects of exogenous variables, and *ζ* is the disturbance term of the equation. In the model, the endogenous vector η includes perceived efficiency, AI concerns, and intention to use AI, while the exogenous vector ξ includes AI knowledge and AI application capability.

This study analyzed a path model within the SEM framework. Accordingly, the results should be interpreted as a path-analytic SEM using observed variables, rather than as a full latent-variable SEM. This approach was employed to examine theoretically specified structural pathways and their regional differences.

All constructs in the analysis were specified in the model as observed composite indices. Each construct (AI knowledge, AI application capability, perceived efficiency, AI concerns, and the intention to use AI) was operationalized using five scale items, and a composite index was created by averaging the corresponding scores. These averaged indices were entered into the model as observed variables rather than as latent factors using a separate measurement model. In the final specification, AI knowledge and AI application capability were treated as exogenous variables, perceived efficiency and AI concerns were specified as perceptual variables in the path model, and the intention to use AI was modeled as the endogenous outcome. Direct paths from AI knowledge and AI application capability to intention to use AI were included to examine whether the associations were consistent with a partial mediation structure, and all equations were additionally controlled for demographics such as age, gender, education, and income. The structural pathways in the conceptual framework are shown in Figure 1.

Among several alternative model specifications, the one with the most favorable balance between explanatory power and simplicity was selected; the retained model exhibited an adequate overall fit to the observed data (RMSEA = 0.068, TLI = 0.915, CFI = 0.996, SRMR = 0.010) and was used for inference in the following sections. The model was estimated using maximum likelihood, and conventional maximum-likelihood standard errors were used. The full model-fit test showed χ^2^(2) = 6.685 (*p* = 0.035).

### 3.2 Data

The dataset was constructed from online survey data. The survey was conducted through a professional online survey panel, and anonymized data without personally identifiable information were used for the analysis. The survey provider managed informed consent, recruitment, and data anonymization according to its standard procedures. The dataset contained no missing values for the variables included in the model. The survey was conducted in Korea in September 2025, using quota sampling by region, gender, and age. The megacity region is defined as the capital area centered in Seoul, whereas non-megacity regions comprise all other administrative regions in Korea. This distinction is useful for examining whether the cognitive pathways linking AI knowledge and AI application capability to the intention to use AI differ between regions with relatively advanced technological conditions and those with relatively limited conditions. The sample covers both AI users and non-users. Thus, this study reflects the cognitive pathways of the general public rather than a specific group.

According to the results of the descriptive statistical analysis (Table 1), male respondents and female respondents accounted for 50% of the sample. In megacity regions, female respondents (51.69%) slightly outnumbered male respondents (48.31%), whereas in non-megacity regions, male respondents (53.14%) accounted for a higher proportion than female respondents (46.86%). Age was relatively evenly distributed across the 20s to 50s age range. As for monthly income, the largest group was the one with an income between KRW 3 and 4 million (14.7%), followed by KRW 2–3 million (13.1%), KRW 4–5 million (12.1%), and KRW 5–6 million (12.0%), indicating a relatively even income distribution. Regarding educational attainment, the majority of respondents (71.4%) were college graduates.

**Table 1:** Descriptive statistics of respondents.

Variable		Megacity		Non-megacity		Total	
		Freq.	%	Freq.	%	Freq.	%
Gender	Male	314	48.31	186	53.14	500	50.00
	Female	336	51.69	164	46.86	500	50.00
Age group	20s	157	24.15	93	26.57	250	25.00
	30s	155	23.85	95	27.14	250	25.00
	40s	172	26.46	78	22.29	250	25.00
	50s	166	25.54	84	24.00	250	25.00
Monthly income (KRW)	< 1 million	17	2.62	16	4.57	33	3.30
	1–2 million	21	3.23	13	3.71	34	3.40
	2–3 million	71	10.92	60	17.14	131	13.10
	3–4 million	93	14.31	54	15.43	147	14.70
	4–5 million	77	11.85	44	12.57	121	12.10
	5–6 million	70	10.77	50	14.29	120	12.00
	6–7 million	74	11.38	32	9.14	106	10.60
	7–8 million	63	9.69	27	7.71	90	9.00
	8–9 million	46	7.08	22	6.29	68	6.80
	9–10 million	41	6.31	10	2.86	51	5.10
	≥ 10 million	77	11.85	22	6.29	99	9.90
Education level	Middle School or Below	2	0.31	2	0.57	4	0.40
	High School	91	14.00	67	19.14	158	15.80
	University Graduate	474	72.92	240	68.57	714	71.40
	Graduate +	83	12.77	41	11.71	124	12.40

The total sample consisted of 1,000 respondents, including 650 from the megacity region and 350 from the non-megacity region. In the model, age and income were treated as continuous variables. Gender was coded 1 = male and 0 = female; education was coded 1 = college degree or higher and 0 = otherwise.

Variables related to the perception of AI were measured based on a five-point Likert scale. The items for each variable consist of five subitems. Items regarding AI knowledge assess the understanding of general AI concepts and principles. Items regarding AI application capability assess the ability to apply AI to problem-solving. Additionally, items regarding perceived efficiency evaluate the perceived ability to collaborate with AI in performing tasks, and items regarding AI concerns assess respondents’ concerns about AI during the acceptance process. Additionally, items regarding intention to use AI indicate the willingness to use AI in the future.

AI knowledge was measured using indicators related to understanding the conceptual differences between AI and general-purpose computer programs, understanding how AI learns from data, recognizing its computational operating principles, understanding its limitations and potential errors, and awareness of its applications across different fields. AI application capability was measured using indicators reflecting proficiency with AI tools, the ability to solve problems with AI, the ability to review and refine AI-generated outputs, the ability to explain the differences between artificial and human intelligence, and adaptability to newly developed AI technologies. Perceived efficiency was assessed using indicators related to task speed processing, ease of task performance, usefulness for idea generation, effectiveness in problem solving, and ease of use. AI concerns were measured using indicators reflecting concerns regarding the accuracy and reliability of AI outputs, privacy and ethical issues, the cost burden, psychological discomfort with human replacement, and job displacement anxiety. Finally, the intention to use AI was measured using indicators of willingness to actively use AI in study or work, to try newly introduced AI technologies, to recommend AI to others, to pay for AI if needed, and to shift from existing work methods to AI-based approaches.

The descriptive statistics, reliability coefficients, and correlations among the constructs are presented in Table 2. Since Cronbach’s *α* values ranged from 0.66 to 0.86, the internal consistency across the constructs was considered acceptable. The correlation between AI knowledge and AI application capability was 0.70, suggesting that the two constructs are related but not redundant. AI knowledge captures respondents’ understanding and awareness of AI, whereas AI application capability captures their perceived ability to use AI in practical contexts. Therefore, the two constructs were treated as related but distinct dimensions in the subsequent path model. To assess reliability comparability across groups, Cronbach’s α values were also calculated separately for megacity and non-megacity respondents. Since Cronbach’s α values ranged from 0.63 to 0.85 in the megacity group and from 0.71 to 0.87 in the non-megacity group, the reliability patterns were considered broadly comparable.

**Table 2:** Descriptive statistics, reliability, and correlations of constructs.

Construct	Mean	SD	Cronbach’s α		Pearson correlation coefficients				
			Full	Group	(1)	(2)	(3)	(4)	(5)
AI knowledge	3.71	0.60	0.81	0.80 / 0.81	1.00				
AI application capability	3.42	0.64	0.86	0.85 / 0.87	0.70	1.00			
Perceived efficiency	3.80	0.59	0.86	0.85 / 0.86	0.49	0.59	1.00		
AI concerns	3.50	0.59	0.66	0.63 / 0.71	0.13	0.07	0.02	1.00	
Intention to use AI	3.69	0.61	0.85	0.84 / 0.85	0.47	0.55	0.67	−0.01	1.00

The Group column indicates group-specific Cronbach’s α values in the order megacity / non-megacity. (1) AI knowledge; (2) AI application capability; (3) Perceived efficiency; (4) AI concerns; (5) Intention to use AI.

## 4 Results

In this study, SEM was used to examine the effects of perceived efficiency and concerns regarding AI on individuals’ intentions to use AI, with a particular focus on the differences in perception pathways between megacity and non-megacity regions. The analytical model specified AI knowledge and application capability, and demographic characteristics, such as age, gender, education, and income, were used as explanatory variables. Based on these model specifications, perceived efficiency and AI concerns were estimated. Then, these two perception variables were included as explanatory variables in the model for intention to use AI, thereby identifying the pathways between cognition and behavior. Analyses were conducted using the same sample, dividing respondents by region to enable comparison and interpretation of path coefficients between megacity and non-megacity regions.

The results of the analysis for the perceived efficiency model are presented in Table 3. This model shows that perceived efficiency is closely associated with individuals’ AI knowledge and application capability, whereas the effects of demographic variables are limited. AI application capability showed the largest positive association in both regions. In the megacity region, the coefficient was 0.464, and in the non-megacity region, it was 0.402, both of which are statistically significant at the 1% level. This result indicates that AI application capability is closely associated with perceived efficiency; the higher the level of hands-on experience and skill, the more strongly individuals perceive AI as enhancing work efficiency beyond mere conceptual understanding.

**Table 3:** Perceived efficiency model.

Category	Variable	Megacity		Non-megacity	
		Coef.	SE	Coef.	SE
Perceived efficiency	AI knowledge	0.094**	0.044	0.259***	0.057
	AI application capability	0.464***	0.043	0.402***	0.054
Demographics	Age	−0.002	0.002	0.002	0.002
	Gender	−0.030	0.037	−0.031	0.050
	Education	0.018	0.054	−0.055	0.064
	Income	0.013*	0.007	−0.005	0.010
	Constant	1.864***	0.157	1.491***	0.191

*, **, and *** indicate significance at the 0.1, 0.05, and 0.01 levels, respectively.

In contrast, AI knowledge exhibited distinct regional differences. In the megacity region, the coefficient was relatively small (0.094). In contrast, in the non-megacity region, the coefficient was more than twice as high (0.259), indicating that the association between AI-related understanding and perceptions of efficiency is stronger outside the megacity region. This finding can be interpreted as reflecting that, due to lower accessibility and familiarity with AI concepts and principles, simply knowing about AI is closely linked to improved perceptions of efficiency in non-megacity regions. In contrast, because a certain level of AI knowledge has already been generalized in the megacity region, practical skills, rather than conceptual knowledge, are more closely related to efficiency perception. Thus, in non-megacity regions, knowledge diffusion may function as a starting point for the formation of perceived efficiency, operating alongside AI application capabilities.

Demographic variables show no clear directional association with perceived efficiency. Age, gender, and education were not significant in either region, whereas income showed a weak positive relationship only in the megacity regions. Thus, the formation of perceived efficiency appears to be capability-centered, with knowledge playing a supplementary role in non-megacity regions. This suggests that the regional gap in AI use arises not only from differences in technical proficiency but also from disparities in awareness and access to information.

The AI concerns model (see Table 4) shows that, unlike efficiency, demographic characteristics, particularly gender and education, have more distinct associations than technical variables. AI knowledge had a modest but significant positive association in both regions. This implies that the more individuals recognize the limitations of AI, such as incorrect information generation, potential errors, privacy violations, data leaks, ambiguous sources, and copyrights, the more likely they are to report higher levels of AI concerns. This pattern indicates that AI knowledge may be associated with concerns in multiple ways. Although greater knowledge can reduce uncertainty, it may also increase awareness of AI-related risks, thereby being associated with higher AI concerns. In other words, such AI concerns may not stem from ignorance but rather from greater awareness of algorithmic limitations and automation threats.

**Table 4:** AI concerns model.

Category	Variable	Megacity		Non-megacity	
		Coef.	SE	Coef.	SE
AI concerns	AI knowledge	0.124**	0.052	0.154**	0.074
	AI application capability	−0.058	0.051	0.055	0.071
Demographics	Age	0.002	0.002	−0.001	0.003
	Gender	−0.198***	0.044	−0.181***	0.064
	Education	−0.132**	0.064	0.008	0.083
	Income	0.004	0.008	−0.013	0.012
	Constant	3.345***	0.187	2.960***	0.248

** and *** indicate significance at the 0.05 and 0.01 levels, respectively.

The AI application capability was not statistically significant in either region, indicating that the degree of technical proficiency was not systematically related to the level of AI concerns. In other words, being skilled in AI does not necessarily reduce or increase AI concerns; instead, it tends to depend on perceptions of risks, such as errors, information reliability, and data protection.

Among the demographic variables, gender was the most consistent and powerful explanatory factor. In the megacity (−0.198) and non-megacity (−0.181) regions, male respondents exhibited significantly lower levels of AI concern than female respondents. This finding is consistent with previous research suggesting that gender differences in AI adoption are closely related to beliefs and barriers regarding AI use (Carvajal et al., 2025; Humlum and Vestergaard, 2025).

This may reflect the differences in technological familiarity, occupational characteristics, and emotional receptivity to AI. Meanwhile, education was significant only in the megacity region, showing a negative effect (−0.132, S. E. = 0.064), indicating that individuals with higher educational attainment experienced lower levels of AI concerns. Education level was not a significant factor in non-megacity regions. This can be interpreted as college graduates from the megacity region having a more positive perception of AI’s practical utility and application potential of AI or a higher adaptability to technological change. Age and income were not statistically significant in either region.

Previous analyses examined the factors associated with perceived efficiency and AI concerns. Subsequently, the effects of these perception variables on the intention to use AI were analyzed. The intention to use AI model (see Table 5) revealed that perceived efficiency showed the strongest association. The higher the perceived efficiency, the stronger the intention to use AI, a trend consistently observed in both regions. The coefficients are 0.540 for megacity regions and 0.552 for non-megacity regions, both of which are statistically significant at the 1% level. Although the difference between the two coefficients is small, the slightly higher value in the non-megacity region is noteworthy. This result indicates that positive perceptions of efficiency are closely associated with intention to use AI across regions, showing that individuals’ recognition of AI’s practical usefulness is closely linked to behavioral intention.

**Table 5:** Intention to use AI model.

Category	Variable	Megacity		Non-megacity	
		Coef.	SE	Coef.	SE
Intention to use AI	Perceived efficiency	0.540***	0.036	0.552***	0.052
	AI concerns	−0.035	0.031	−0.046	0.040
	AI knowledge	0.067*	0.041	0.130**	0.057
	AI application capability	0.199***	0.043	0.127**	0.056
Demographics	Age	0.003*	0.002	−0.001	0.002
	Gender	0.063*	0.035	0.047	0.048
	Education	0.054	0.050	0.086	0.062
	Income	0.003	0.006	−0.002	0.009
	Constant	0.629***	0.193	0.788***	0.231

*, ** and *** indicate significance at the 0.1, 0.05 and 0.01 levels, respectively.

In contrast, AI concerns show a limited association with the intention to use AI. Although the coefficients for both regions are negative, they are not statistically significant. This finding indicates that AI concerns were not strongly associated with lower intention to use AI. As AI provides significant convenience and practical benefits, existing concerns or negative perceptions may not be sufficient to suppress the intention to use it. Previous studies have repeatedly confirmed that perceptions of usefulness and convenience outweigh conflicts or resistance during technology adoption (Balakrishnan et al., 2024; Lyu et al., 2025). Furthermore, growing societal expectations of AI technologies and institutional encouragement appear to have limited the extent to which critical awareness translates into behavioral restraint. The effects of demographic variables were limited. Education and income were not significant in either region, whereas age and gender were weakly significant only in the megacity region.

To examine regional differences between megacity and non-megacity groups, Wald tests of equality constraints on path coefficients were conducted (see Table 6). The results show that a significant regional difference was observed in the path from AI knowledge to perceived efficiency, where the coefficient was larger in the non-megacity region. Other paths, including the direct paths from AI knowledge and AI application capability to intention to use AI, did not significantly differ across regions.

**Table 6:** Tests of regional differences in path coefficients.

Path	Coefficients		Wald χ²	*p*-value
	Megacity	Non-megacity		
AI knowledge → Perceived efficiency	0.094	0.259	5.285	0.022
AI application capability → Perceived efficiency	0.464	0.402	0.796	0.372
AI knowledge → AI concerns	0.124	0.154	0.112	0.738
AI application capability → AI concerns	−0.058	0.055	1.694	0.193
Perceived efficiency → Intention to use AI	0.540	0.552	0.037	0.847
AI concerns → Intention to use AI	−0.035	−0.046	0.052	0.819
AI knowledge → Intention to use AI	0.067	0.130	0.799	0.371
AI application capability → Intention to use AI	0.199	0.127	1.049	0.306

The equality of corresponding path coefficients across regional groups was tested. Coefficients are unstandardized estimates from models controlling for age, gender, education, and income. df = 1 for all tests.

Based on the SEM framework, both the direct and indirect effects on the intention to use AI were analyzed (see Table 7). Perceived efficiency exhibited the strongest direct effect in both regions, showing a consistent positive association with intention to use AI. In contrast, AI concerns showed a small, statistically insignificant direct effect, suggesting a limited role in explaining the intention to use AI.

**Table 7:** Effect decomposition from the path model.

Category	Variable	Megacity			Non-megacity		
		Direct	Indirect	Total	Direct	Indirect	Total
Intention to use AI	Perceived efficiency	0.540***		0.540***	0.552***		0.552***
	AI concerns	−0.035		−0.035	−0.046		−0.046
Demographics	AI knowledge	0.067*	0.046*	0.114**	0.130**	0.136***	0.266***
	AI application capability	0.199***	0.252***	0.451***	0.127**	0.219***	0.346***
	Age	0.003*	−0.001	0.002	−0.001	0.001	0.000
	Gender	0.063*	−0.009	0.054	0.047	−0.009	0.038
	Education	0.054	0.014	0.069	0.086	−0.031	0.055
	Income	0.003	0.007*	0.010	−0.002	−0.002	−0.004

*, **, and *** indicate significance at the 0.1, 0.05, and 0.01 levels, respectively.

AI knowledge and application capability are expected to be indirectly associated with intention to use AI through perceived efficiency and AI concerns. Both variables exert significant positive indirect effects on the intention to use AI. AI knowledge showed a larger indirect effect in non-megacity regions. Its indirect effect was relatively low in the megacity region (0.046) but nearly three times higher in the non-megacity region (0.136). This suggests that in the non-megacity region, understanding AI concepts is strongly associated with perceived efficiency, which in turn is associated with the intention to use AI. This result is consistent with earlier findings that conceptual understanding shows a stronger association with efficiency perceptions in non-megacity regions, suggesting that access to technology and information availability is more closely related to behavioral intention in contexts outside megacity regions.

Conversely, in megacities, AI application capability has emerged as a major associated factor. The indirect effect is substantial in both regions (0.252 and 0.219 in the megacity and non-megacity regions, respectively). This suggests that, in both regions, practical proficiency in handling AI is associated with perceived efficiency, which in turn is linked to a stronger intention to use AI. In megacity regions, the association involving capability was greater than that of conceptual understanding, suggesting that hands-on experience and technical skills played more direct roles in shaping intentions.

In the non-megacity region, the level of understanding of AI and practical application capability are closely associated with perceived efficiency. In contrast, in the megacity region, AI application capability is especially important, as it is directly associated with the intention to use AI and has an indirect effect through perceived efficiency. This pathway pattern indicates that the regional gap in AI use arises from differences in proficiency and disparities in basic awareness and information environments.

The direct and indirect effects of demographic variables were weak. However, in the megacity region, income showed a modest indirect effect through perceived efficiency, and the total effect was statistically significant. In contrast, in non-megacity regions, variables including income were not significant.

In summary, the results indicated that perceived efficiency, AI application capability, and AI knowledge are the primary factors associated with intention formation, while sociodemographic characteristics are secondary factors. This finding may suggest that to address the regional gap in AI adoption, policies should place greater emphasis on mitigating regional differences in perceptions and capabilities rather than focusing solely on socioeconomic inequality.

## 5 Discussion

### 5.1 Theoretical and empirical implications

The traditional TAM emphasizes the individual-level pathway from perceived usefulness to the behavioral intention to use (Davis, 1989). However, the results regarding the intention to use AI revealed a multifaceted pattern. These results are consistent with the theoretically specified partial mediation structure: AI knowledge and application capability are associated with the intention to use AI not only indirectly through perceived efficiency but also directly. Therefore, the decision to adopt AI is associated with both perceived efficiency and individuals’ sense that they understand AI and can apply it in practice. This finding is broadly consistent with prior TAM-based studies showing that positive evaluations of usefulness and ease of use are central to AI adoption (Na et al., 2022; Khan et al., 2024). However, the present study extends this literature by showing that the pathway to intention is not limited to perceptions of AI alone but is also related to the regionally differentiated effects of AI knowledge and AI application capability.

In addition, this study suggests that such a pathway does not manifest uniformly across contexts. In the megacity region, the path from AI application capability through perceived efficiency to the intention to use AI appears to be the strongest. In a non-megacity region, both AI knowledge and application capability are associated with perceived efficiency, which in turn is linked to the intention to use AI. In addition, both factors were directly associated with the intention to use AI. A regional comparison of these models suggests that the major factors associated with the perception of AI as an efficiency enhancer differ in their cognitive pathways across regions. This divergence reflects not a mere difference in level but rather a difference in mechanism. This result aligns with digital divide research, suggesting that differences in access, familiarity, and information resources affect the extent of technology use (Hargittai, 2001; Helsper, 2021). At the same time, our findings extend this perspective by showing that regional disparities are reflected not only in overall levels of technological readiness but also in the specific cognitive pathways through which AI knowledge and application capability are linked to the intention to use AI. In a megacity region, the ability of individuals to apply AI in practice is associated with their perceived efficiency, which in turn is linked to their intention to use AI. Thus, in megacity regions, the belief that AI enables faster and more accurate work serves as an important factor associated with decision-making. In contrast, in the non-megacity region, understanding how AI is associated with perceived efficiency means that basic literacy and awareness—rather than advanced application skills—become important factors associated with decision-making. In other words, it helps people grasp how AI can function as a foundational condition for forming perceived efficiency. This interpretation is consistent with the digital divide perspective, emphasizing that conceptual access to technology is unevenly distributed across contexts (Helsper, 2012; Suh, 2025).

From a policy perspective, this suggests that the strategies may need to differ by region. In megacities, measures that strengthen practical AI application capabilities and reinforce perceived efficiency–for instance, by demonstrating productivity gains and ensuring reliability and error control–can be more relevant to fostering the intention to use AI. In other words, promoting human–AI synergistic workflows is likely to be an important motivational factor. In contrast, for non-megacity regions, basic education and raising awareness of AI need greater emphasis. Consequently, a one-size-fits-all approach to AI promotion may be limited. Policies to encourage AI adoption and utilization should consider region-specific differences and each region’s distinct motivational and cognitive frames.

### 5.2 Decision-making and policy implications

This study hypothesized that perceived efficiency would positively influence individuals’ intentions to use AI, whereas AI concerns would negatively affect these intentions. Although the effect of perceived efficiency was positive and significant, the effect of AI concerns was weak or statistically insignificant. AI-related concerns can coexist with positive perceptions of AI’s efficiency benefits (Gillespie et al., 2023). Rather than perceiving AI in strictly dichotomous terms as either positive or negative, individuals may accept AI through a risk–benefit evaluation that weighs perceived benefits against perceived risks (Brauner et al., 2025). The results of this study suggest a close association between perceived efficiency and AI acceptance, whereas AI concerns show only a limited direct association with intention to use AI. In other words, even when people harbor concerns about AI, the belief that AI enhances performance serves as an important basis for their intention to use it. This pattern is consistent with prior studies, suggesting that perceived usefulness or convenience often outweighs resistance- or risk-related perceptions of technology adoption (Balakrishnan et al., 2024; Lyu et al., 2025).

From a decision-making perspective, this suggests that perceived efficiency may be closely associated with AI acceptance. Particularly in the regional context, among groups in the megacity region that already possess a certain level of AI application capability, expectations about improved work performance show a stronger association with intention to use AI than concerns about AI. This pattern suggests that benefit-related perceptions may be more salient than risk-related perceptions in shaping AI acceptance.

However, the analysis does not include measured data on actual productivity or learning outcomes, nor does it examine policy intervention effects. Therefore, the intention to use AI should not be equated with actual adoption behavior, and these policy implications should be viewed as cautious considerations rather than definitive policy recommendations. It is also important to note that AI policy should not be designed solely to encourage more active AI use. Alongside the potential benefits of AI adoption, policymakers should carefully consider risks related to unequal access, misinformation, and the reinforcement of existing inequalities (Capraro et al., 2024). Moreover, when AI outputs are accepted without sufficient verification, they may create epistemic risks in decision-making contexts. Thus, efforts to strengthen AI capabilities should include training in critical evaluation and responsible use (Loru et al., 2025; Quattrociocchi et al., 2025).

In the models, estimations were conducted controlling for demographic and socioeconomic characteristics, including income. Thus, regional contrasts could be interpreted as differences in how people cognitively link AI readiness to behavioral intention rather than as simple consequences of socioeconomic inequality. In short, regional gaps are manifested in cognitive and experiential aspects, not simply economic ones.

## 6 Conclusion

This study analyzed how individuals’ cognitive and decision-making pathways regarding AI are associated with their intention to use it by estimating three models: the perceived efficiency model, the AI concerns model, and the intention to use AI model. It then identifies how these pathways differ between megacity and non-megacity regions. AI knowledge and application capability are established as exogenous variables, whereas perceived efficiency and AI concerns are included as perceptual variables within the SEM framework. A comparative analysis was conducted by separating respondents into megacity and non-megacity groups.

The analysis revealed that perceived efficiency was the factor most strongly associated with intention to use AI in both regions. The higher the perceived efficiency, the stronger the intention to use AI, consistently suggesting that the recognition of the practical usefulness of technology plays a central role in shaping behavioral intentions. By contrast, AI concerns did not have a statistically significant effect on the intention to use AI, indicating that even when individuals experience certain anxieties or negative perceptions, AI’s convenience and utility may remain more closely linked to intention to use AI.

Furthermore, the effects of AI knowledge and application capability on perceived efficiency differ across regions. In the megacity region, the ability to use AI (application capability) showed a pronounced effect on perceived efficiency, indicating that hands-on proficiency and direct experience with AI are key factors associated with efficiency recognition. By contrast, in the non-megacity region, both AI application capability and understanding AI concepts (AI knowledge) were associated with perceived efficiency, and the effect of basic AI knowledge was comparatively stronger. In other words, outside the megacity region, gaining familiarity with AI and what it can do is associated with higher perception of efficiency. That is, AI application capability serves as a central factor associated with perceived efficiency. In the megacity region, the variance in knowledge level is relatively low, making capability a stronger differentiator. In the non-megacity region, the diffusion of conceptual understanding itself becomes the starting point for efficiency recognition. These findings suggest that a single cognitive pathway cannot explain AI acceptance. Rather, the pathways of perception formation differ depending on the regional information environment and the level of experience.

However, the effect of sociodemographic characteristics on intention to use AI is limited. Age, income, and education were mostly insignificant, whereas in megacity regions, male respondents showed a slightly higher intention to use AI. This may reflect social and cultural factors such as familiarity with technology or occupational characteristics. Overall, the intention to use AI was more strongly explained by cognitive factors (i.e., perception and capability) than by socioeconomic background. These patterns indicate that the observed regional differences reflect distinct cognitive pathways, rather than a simple socioeconomic composition.

These results have theoretical and policy implications. First, they extend the traditional TAM by presenting a pathway framework of perception, concern, and behavior that connects AI knowledge and application capabilities to perceptual factors, thereby expanding the theoretical scope of AI acceptance research. While prior TAM-based AI studies have mainly emphasized general acceptance factors within particular technological applications, this study suggests the dual relevance of AI knowledge and application capability, showing that these factors are associated with both perceived efficiency and AI concerns. Second, the pathway of AI adoption is not uniform across sociospatial contexts. By comparing megacity and non-megacity regions, this study empirically shows how contextual conditions are associated with technology acceptance. It shows that the relative importance of knowledge- and capability-based pathways may vary across regional contexts, proposing a regionally contextualized approach to technology acceptance research. Third, from a policy perspective, the findings suggest that regional gaps in the intention to use an AI should not be perceived merely as a problem of technical proficiency but rather as a difference in perception and the informational environment. In megacities, rather than focusing solely on practice-based skill enhancement, greater emphasis could be placed on improving AI ethics, trust, and data literacy. In contrast, in non-megacity regions, greater emphasis could be placed on strengthening basic AI understanding and expanding education and infrastructure to improve accessibility.

However, the findings of this study should be interpreted cautiously. Unequal sample sizes across regional groups may result in reduced statistical precision. Furthermore, online surveys may underrepresent the circumstances of less digitally active individuals. Since cross-sectional data was used, it is difficult to fully identify changes in perception over time or establish causal directions. Moreover, the classification was simplified to a binary distinction between megacities and non-megacities. The non-megacity group includes heterogeneous regional contexts, such as large provincial cities, smaller cities, and less urbanized areas. Thus, the megacity/non-megacity classification should be interpreted as a broad regional contrast rather than as a precise measure of urbanicity or technological access. To discuss the contextual effects more rigorously, it is necessary to consider more fine-grained groups, such as small cities or rural settings, in future research. Finally, to analyze the acceptance pathways more precisely in specific contexts, variables related to AI experience or job relevance should be incorporated into future models.

Nevertheless, this study still remains significant in that it empirically examined regional differences in cognitive pathways toward AI, amidst the growing importance of AI. By comparing the pathway relationships among perceived efficiency, AI concerns, and the intention to use AI, this study provides implications for how environmental disparities are associated with the cognitive mechanisms underlying AI acceptance. These results can improve our understanding of the conditions for a desirable digital transition by highlighting the need for regionally tailored cognitive and informational strategies to ensure balanced AI diffusion.

## Data Availability

The raw data supporting the conclusions of this article will be made available by the authors, without undue reservation.
